# A follow-up study on the quality of alcohol dependence-related information on the web

**DOI:** 10.1186/1747-597X-6-13

**Published:** 2011-06-10

**Authors:** Olivier Coquard, Sebastien Fernandez, Daniele Zullino, Yasser Khazaal

**Affiliations:** 1 Department of Psychiatry, University Hospital of Vaud, Lausanne, Switzerland; 2 Geneva University, Geneva, Switzerland; 3 Division of Substance Abuse, Geneva University Hospitals, Geneva, Switzerland

**Keywords:** Internet, Quality Indicators, Health Care, alcohol, addiction

## Abstract

In order to evaluate the one-year evolution of web-based information on alcohol dependence, we re-assessed alcohol-related sites in July 2007 with the same evaluating tool that had been used to assess these sites in June 2006. Websites were assessed with a standardized form designed to rate sites on the basis of accountability, presentation, interactivity, readability, and content quality. The DISCERN scale was also used, which aimed to assist persons without content expertise in assessing the quality of written health publications. Scores were highly stable for all components of the form one year later (*r *= .77 to .95, *p *< .01). Analysis of variance for repeated measures showed no time effect, no interaction between time and scale, no interaction between time and group (affiliation categories), and no interaction between time, group, and scale. The study highlights lack of change of alcohol-dependence-related web pages across one year.

## Background

Health issues are among the most searched topics on the web [1]. For this reason, it is particularly important for websites to present high-quality, accurate information on health-related topics such as alcohol dependence.

However, general concern has been expressed about the quality of web-based health information designed for consumers [2,3]. This finding holds most frequently true in the area of addiction-related disorders [4-6], including alcohol dependence [7,8]. Most available studies have a cross-sectional design and little is known about the evolution of the quality of mental health and addiction-related websites over time. In consideration of the lack of quality observed in cross-sectional studies, it is important to assess whether websites improve spontaneously by one-year follow-up (a reasonable period to improve a website). One previous study [9] assessing the evolution of suicide prevention websites following assessment and feedback sent to website administrators concluded that feedback did not lead to improvement of website content.

There is a general agreement about the characteristics of a good health-related website [10,11]. These characteristics include quality of content (evidence-based information), design and aesthetics of the site, readability, dating of information, authority of source, ease of use, accessibility, and disclosure of authors and sponsors.

The present study aimed to assess the evolution of a sample of alcohol-related websites between June 2006 and July 2007.

## Methods

A web search was performed that aimed to produce a list of websites similar to one that would be generated by a hypothetical French-speaking person with limited medical or internet knowledge. Keyword searches were done in June 2006 [8] to identify websites providing information on alcohol addiction in the French language. The standard keywords (in French) "alcoholism" and "alcohol dependence" were entered into three popular search engines: Google, Yahoo, and MSN.

The first 20 websites returned from each keyword query were examined for study inclusion, as most people rarely search beyond the first 20 retrieved links [2]. Prior studies [2] show that more than 95% of people searching for medical information on the internet most often explore the first 10 links, whereas less than 5% explore links that rank between 10 and 20.

Exclusion criteria of websites were as follows: having an invalid address, having been previously reviewed in the present study, containing no information on alcohol addiction or abuse, requiring an access fee, being a discussion group or open forum, not being a site (external links, books, or articles), and having no information in French. We reviewed 120 websites. There was a sizeable overlap among the sites identified by the different search engines and the two keywords (32/110). This left 88 websites, from which 43 were further excluded for the following reasons: 19 contained no information on alcohol dependence; 1 required an access fee; 11 were discussion groups or open forums; 12 were only external links or books. Forty-five websites were included and analyzed in June 2006.

In the present study, the 45 websites evaluated in the original study were searched to see whether they still existed and if so, whether their quality had been modified. Sites were assessed by using the same scoring system applied in the original study and in other previously published works [4,12].

Website affiliations were divided into five categories--commercial, university, non-profit organization, governmental, or other--according to the suffix and the declared affiliation (.gov: government; .edu: university; .com: commercial; .org: non-profit organization).

A standardized form, based on previous studies [10,13-17], was adapted to avoid overlap between instruments (i.e., between Silberg and Abbott scales), containing the following sections:

1. Silberg scale [10,14]: This instrument assessed accountability based on criteria of authorship (whether authors and their affiliations and credentials were identified), attribution (whether sources and references were mentioned), disclosure (whether ownership of the site, sponsoring, and advertising were disclosed), and currency (whether the date of creation and modification of the site has been specified).

2. Interactivity: This was assessed with an adaptation of the Abbott scale [13], which evaluates the presence of a within-site search engine, audio or video support, evaluation questionnaires for users, supporting bodies (forums), and the option to send questions to the webmaster or authors.

3. Aesthetic criteria: These issues are evaluated with Abbott's criteria [13], adapted by Kisely et al. [16], covering the presence of headings, subheadings, diagrams, and hyperlinks, as well as the absence of advertising.

4. Readability was assessed by using the Flesch-Kincaid Grade Level score and the Flesch-Kincaid Readability Index [16]. The first score evaluates the degree of text reading difficulty in regard to USA school grades. Higher scores reflect higher levels of difficulty. A score of 8, the recommended level for standard documents, means that an eighth grade student could understand the text. The second score [16] is included in the Microsoft Word spellchecker and ranges from 0 to 100, with higher scores reflecting higher legibility. Readability scores were calculated with mathematical formulas that treated the number of words, sentences, and syllables.

5. Content quality: This refers to the evidence-based quality of the information and was assessed from the availability of responses to probable queries. The author's choice of question types focused on advice for treatment and information concerning diseases, as these are frequent queries on the internet [18]. The retrieved information was compared with official guidelines (American Psychiatric Association, 2006: "Practice Guidelines for the Treatment of Substance Use Disorders"). The following questions were assessed: 1) definition of alcohol addiction and alcohol abuse, 2) somatic complications, 3) psychosocial complications of alcohol addiction and abuse, 4) withdrawal treatments, 5) psychological treatment, and 6) maintenance treatments. Coverage and correctness of medical information were evaluated. The coverage of a topic was characterized as "none", "minimal", and "sufficient" (0-2 points). Correctness of information was characterized as "mostly not", "mostly", and "completely right" (0-2 points). The content quality score for a given site was defined to correspond to the sum of exhaustibility and accuracy for the six studied aspects, amounting to a maximum total of 24 points.

6. Global score: As previously described, a global score was calculated as the sum of Silberg, interactivity, Abbott's aesthetic criteria, and content quality [14,15].

7. DISCERN: The DISCERN scale was used, which assists people without content expertise to assess the quality of written health attributes of a publication, such as the extent to which the information appears balanced and unbiased [19,20]. The instrument comprises 16 items (each rated from 1 to 5). An association was previously found between content quality and DISCERN scores in most [20-22], though not all, studies [4,12].

Inter-rater reliability of scores was assessed from a random sample of sites with two evaluators and resulted in a good inter-rater reliability for all items: Silberg (*r *= .841; *p *< .05), Flesch-Kincaid readability ease (*r *= .881; *p *< .05), Flesch-Kincaid grade level (*r *= .835; *p *< .01), Abbott's aesthetic criteria (*r *= .751; *p *< .05), DISCERN (*r *= .942; *p *< .01), content quality (*r *= .851; *p *< .01), interactivity (*r *= .865; *p *< .01). These findings were similar to those found previously [12].

The sites were assessed by the same psychiatrist and trained evaluator (OC), who was included in the inter-rater reliability assessment and was blind to the detailed scores previously obtained for each website.

Statistical analyses were performed using SPSS for Windows (version 11.0). An initial exploratory analysis involved the calculation of proportions, as well as means and standard deviation of the outcome values. Pearson correlations assessed the test-retest reliability of each component of the form between June 2006 and July 2007. Quality scores were also submitted to a Time (2) × Scale (7) × Affiliation (5) three-way mixed analysis of variance (ANOVA) with repeated measures for the first and second factors. The seven scales described in the Methods section were included in this analysis.

## Results

As shown in Table 1, the overall quality of the sites was relatively poor. For example, at the first assessment, 62.2% of websites had no data on pharmacotherapy for alcohol dependence. For websites with information available on this issue, 17.6% had "mostly not right" information and only 52.9% had "completely right" information.

**Table 1:** Quality indicators of websites, mean (± SD), in June 2006 and July 2007

Score	June 2006 *N *= 45	July 2007 *N *= 38
Silberg scores (0-9)	4.47 (1.91)	4.89 (1.97)

Interactivity scores (0-6)	2.37 (1.22)	2.5 (1.20)

Abbott aesthetic criteria scores (0-4)	2.92 (.82)	2.79 (.87)

Flesch reading ease scores (0-100)	41.99 (12.38)	40.82 (11.53)

Flesch-Kincaid education scores	12.37 (2.75)	12.43 (2.56)

Content quality scores (0-24)	12.82 (6.36)	12.45 (6.94)

Global score (0-43)	22.58 (7.19)	22.63 (7.48)

DISCERN scores (16-80)	47.47 (10.99)	47.16 (10.52)

Thirty-eight of the 45 (84.44%) original sites still existed. Only 55.3% of them reported an update during the last six months. A high test-retest reliability of scores was found for all components of the form (*r *= .77 to .95, *p *< .01; Table 2).

**Table 2:** Correlations between main quality outcome scores between June 2006 and July 2007

Scale (*N *= 38)	Test-retest reliability score
Silberg	.781**

Flesh-Reading Ease	.839**

Flesh Kincaid	.827**

Interactivity	.905**

Aesthetism	.769**

DISCERN	.946**

Content quality	.904**

Global score	.915**

** *p *< .01

At follow-up, the distribution of the site affiliations was similar to that observed in the first evaluation. The percentage of websites in each category and their numbers at the second evaluation were as follows: government: 2.6% (*N *= 1); non-profit organization: 47.4% (*N *= 18); university: 7.9% (*N *= 3); commercial 18.4% (*N *= 7); individual: 21.1 (*N *= 8); unknown: 2.6% (*N *= 1).

By three-way mixed ANOVA for repeated measures, there was no time effect (*F*(1, 32) = 1.587, n.s); no interaction between time and scale (*F*(1, 32) = 1.169, n.s); no interaction between time and group (affiliation categories) (*F *(5, 32) = 1.412, n.s); and no interaction between time, group, and scale (*F *(5, 32) = .748, n.s). The word "scale" here means the seven scales included in the analysis and described earlier.

## Discussion

The present study systematically assessed the evolution of websites across time. Websites showed no statistically significant improvement for any of the outcome variables. One may argue that there have been no major advances during the study period, and so it may be normal to have no major change in these websites. The overall quality of the sites was, however, poor at first assessment and remained poor one year later, despite a real need for improvement, thus showing no spontaneous progress. The present results are in accordance with a previous study [9] that found no improvement in websites assessed at follow-up despite feedback sent to the website managers.

The present study has several limitations. It accounts only for alcohol addiction-related French language internet websites between June 2006 and July 2007. It takes into account the evolution of the same websites and the conclusion cannot be extended to new sites resulting from the same search engine queries one year later (overlap between two similar queries in June 2006 and July 2007: 44.44%). The present study aimed, however, to study the evolution of a group of websites rather than the general evolution linked to specific queries.

This study provides evidence for lack of evolution of alcohol addiction-related web pages across one year and argues for the development of strategies that aim to increase the quality of web-related content.

## Competing interests

The authors declare that they have no competing interests.

## Authors' contributions

OC and YK designed the study. SF conducted the statistical analyses. OC wrote the first draft and compiled the co-authors' suggestions. All authors participated in the drafting of the manuscript and approved the final version.
